# Early-Stage Detection of Ovarian Cancer Based on Clinical Data Using Machine Learning Approaches

**DOI:** 10.3390/jpm12081211

**Published:** 2022-07-25

**Authors:** Md. Martuza Ahamad, Sakifa Aktar, Md. Jamal Uddin, Tasnia Rahman, Salem A. Alyami, Samer Al-Ashhab, Hanan Fawaz Akhdar, AKM Azad, Mohammad Ali Moni

**Affiliations:** 1 Department of Computer Science and Engineering, Bangabandhu Sheikh Mujibur Rahman Science and Technology University, Gopalganj 8100, Bangladesh; martuza.cse@bsmrstu.edu.bd (M.M.A.); sakifa.cse@bsmrstu.edu.bd (S.A.); jamal.bsmrstu@gmail.com (M.J.U.); 2 Department of Computer Science and Engineering, Rajshahi University of Engineering and Technology, Rajshahi 6200, Bangladesh; sruti.cse13.ruet@gmail.com; 3 Department of Mathematics and Statistics, Faculty of Science, Imam Mohammad Ibn Saud Islamic University (IMSIU), Riyadh 13318, Saudi Arabia; saalyami@imamu.edu.sa (S.A.A.); ssashhab@imamu.edu.sa (S.A.-A.); 4 Department of Physics, Faculty of Science, Imam Mohammad Ibn Saud Islamic University (IMSIU), Riyadh 13318, Saudi Arabia; hfakdar@imamu.edu.sa; 5 Faculty of Science, Engineering & Technology, Swinburne University of Technology, Sydney 2150, Australia; aazad@swin.edu.au or; 6 ProCan®, Children’s Medical Research Institute, Faculty of Medicine and Health, The University of Sydney, Westmead, NSW 2145, Australia; 7 School of Health and Rehabilitation Sciences, Faculty of Health and Behavioural Sciences, The University of Queensland, St Lucia, QLD 4072, Australia

**Keywords:** ovarian cancer, benign ovarian tumors, tumor marker, machine learning, statistical analysis

## Abstract

One of the common types of cancer for women is ovarian cancer. Still, at present, there are no drug therapies that can properly cure this deadly disease. However, early-stage detection could boost the life expectancy of the patients. The main aim of this work is to apply machine learning models along with statistical methods to the clinical data obtained from 349 patient individuals to conduct predictive analytics for early diagnosis. In statistical analysis, Student’s *t*-test as well as log fold changes of two groups are used to find the significant blood biomarkers. Furthermore, a set of machine learning models including Random Forest (RF), Support Vector Machine (SVM), Decision Tree (DT), Extreme Gradient Boosting Machine (XGBoost), Logistic Regression (LR), Gradient Boosting Machine (GBM) and Light Gradient Boosting Machine (LGBM) are used to build classification models to stratify benign-vs.-malignant ovarian cancer patients. Both of the analysis techniques recognized that the serumsamples carbohydrate antigen 125, carbohydrate antigen 19-9, carcinoembryonic antigen and human epididymis protein 4 are the top-most significant biomarkers as well as neutrophil ratio, thrombocytocrit, hematocrit blood samples, alanine aminotransferase, calcium, indirect bilirubin, uric acid, natriumas as general chemistry tests. Moreover, the results from predictive analysis suggest that the machine learning models can classify malignant patients from benign patients with accuracy as good as 91%. Since generally, early-stage detection is not available, machine learning detection could play a significant role in cancer diagnosis.

## 1. Introduction

One of the familiar types of malignancy, ovarian cancer (OC), is the seventh most well-known cancer among females, which has a 2.7% lifetime risk factor [1]. Ovarian cancer represents 2.5% of all malignancies among females; however, 5% of the malignant cases die due to low survival rates. This is generally attributed to the late-stage diagnosis and lack of early symptoms [2]. Ovarian cancers are chemo-sensitive, and they show the fundamental adaptability against platinum/taxane treatment, and the recurrence rate is 60–80% within 5 years [3].

Gynecologists typically need to diagnose whether a patient has developed malignant pelvis masses, which can be suspected as tumors [4]. Although a few techniques, e.g., ultrasonography and helical CT scanning, have been utilized to distinguish between a benign tumor and malignant non-gynecologic conditions, the tumor biomarkers such as carbohydrate antigen 125 (CA125), carbohydrate antigen 72-4 (CA72-4) [4], and human epididymis protein 4 (HE4) detection are some of the crucial components in separating female pelvic masses [4,5]. There are some studies that determine the efficiency of those biomarkers in differentiating ovarian cancer and benign tumors. To predict epithelial ovarian cancer, Moore et al. conducted a comparative study between RMI and ROMA algorithms among 457 patients, and they identified that ROMA predicted epithelial ovarian cancer patients with higher sensitivity than RMI [6]. Anton et al. compared the sensitivity of CA125, HE4, ROMA, and RMI among 128 patients and observed HE4 with the highest sensitivity to evaluate the malignant ovarian tumor [7]. Moreover, to predict the progression of ovarian cancer, a multi-marker linear model was developed by Zhang et al. by employing CA125, HE4, progesterone, and estradiol [8].

Machine learning algorithms with novel methodologies have great potentialities in predicting disease progression and malignancy diagnosis. Alqudah et al. used the machine learning algorithms with a wavelet feature selection approach using a serum proteomic profiling dataset [9]. Next, Kawakami et al. performed supervised machine learning classifiers, including GBM, SVM, RF, CRF, Naive Bayes, Neural Network, and Elastic Net using different blood biomarkers to predict the tumor size, but those models achieved only less than 70% AUC score [10]. Paik et al. employed a four-staged OC, histological information, different types of primary treatments, and chemotherapy regimen information, and they predicted the cancer stages with about 83% accuracy score [11]. Recently, Akazawa et al. had performed the machine learning-based analysis with several models such as SVM, Naive Bayes, XGBoost, LR, RF and achieved improved model performance with the XGBoost algorithm with the best accuracy score of around 80% among other competing models [12]. However, this study was sensitive to the size of the feature set; i.e., as the number of feature decreases, the accuracy drops around 60%. Another drawback of this work has been the low number features, i.e., only 16 different blood parameters. Lu et al. used three different types of biomarkers including blood samples, general chemistry medical tests, and OC markers, and they showed a high validation accuracy score but low testing accuracy [5], which indicates the presence of a common problem in machine learning algorithms, i.e., over-fitting. Therefore, a robust framework for stratifying ovarian cancer patients using biomarker features by employing machine learning and statistical analysis is a pressing need at this moment.

We have seen that although multiple studies have been conducted to diagnose ovarian cancer, the accuracy ratings are not adequate, so there is still room for improvement. Additionally, no study has separated the data’s aspects using criteria such as blood samples, general chemistry tests, and OC biomarkers. As a result, we have started with data separation. Only statistical approaches were used in the prior investigation, but we used a mixed methodology (statistical and machine learning) to analyze the data. This method added a new dimension to work and increased the dependability of actual clinical testing, which could benefit clinicians and physicians.

The main objectives of our work are as follows:

- Early-stage detection of ovarian cancer using biomarkers;
- Find the significant and associative biomarkers using statistical methods as well as machine learning models;
- Apply machine learning models on a comprehensive dataset including blood samples, general chemistry medical tests, and OC markers and perform robust and statistically sound analytical experiments.

## 2. Materials and Methods

**Figure 1:**
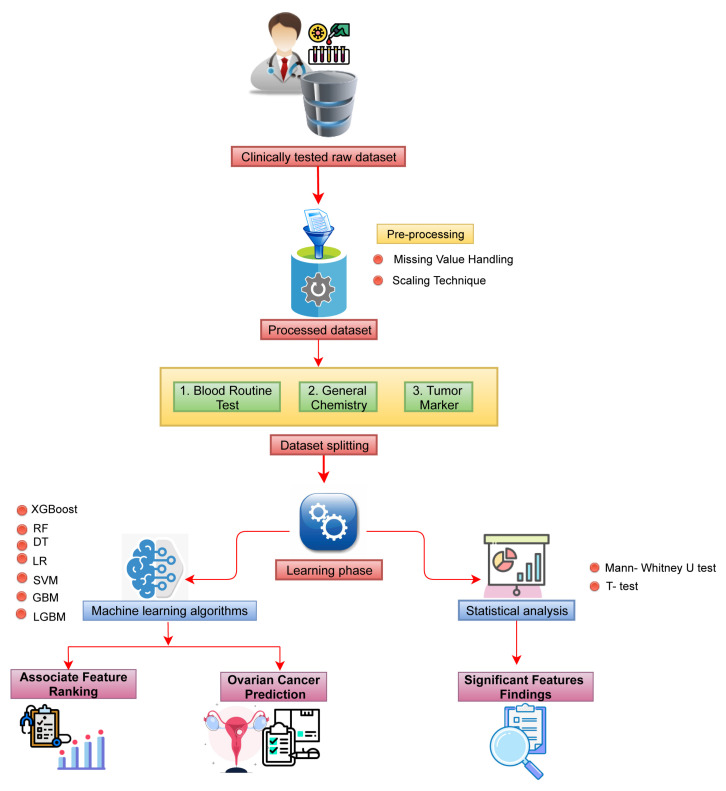
The schematic diagram of the overall workflow.

In this study, we used a clinically tested raw dataset comprised of samples from benign ovarian tumors and malignant ovarian patients. Next, statistical analysis was conducted to identify the most significant biomarkers associated with malignancy. Moreover, the machine learning classification models were employed to detect ovarian cancer in the early stage. The detailed pictorial representation of the workflow is depicted in Figure 1.

### 2.1. Data Collection

The dataset of 349 patients was collected from the ‘Third Affiliated Hospital of Soochow University’ [5]. The retrospective study was taken from July 2011 to July 2018, including 171 ovarian cancer patients and 178 benign ovarian tumor patients. The dataset consists of 49 features that were collected by the pathology diagnosed. We divided the whole dataset into three subgroups: blood routine test (neutrophil ratio, thrombocytocrit, hematocrit, mean corpuscular hemoglubin, lymphocyte, platelet distribution width, mean corpuscular volume, platelet count, hemoglobin, eosinophil ratio, mean platelet volume, basophil cell count, red blood cell count, mononuclear cell count, red blood cell distribution width, and basophil cell ratio), general chemistry (albumin, calcium, indirect bilirubin, uric acid, nutrium, total protein, alanine aminotransderase, total bilirubin, blood urea nitrogen, magnesium, glucose, creatinine, phosphorus, globulin, gama glutamyl tranferasey, alkaline phosphates, kalium, direct bilirubin, carban dioxide-combining power, chlorine, aspartate aminotransferase, and anion gap) and tumor marker (carbohydrate antigen 72-4, alpha-fetoprotein, carbohydrate antigen 19-9, menopause, carbohydrate antigen 125, carcinoembryonic antigen, age, and human epididymic protein 4)) (shown in Table 1). The names of the attributes including some statistical analysis results such as the mean, standard deviation, 95% CI and the *p* values for Student’s *t*-test are shown in Table 2.

### 2.2. Data Processing

The raw dataset was subjected to a series of preprocessing steps, including data cleaning, missing value imputation, data scaling, and data dividing in the preprocessing step. There are 349 individual patients’ information in our dataset, and there were only about 7% of missing values, which were imputed with their mean values of existing values of each features. For the data scaling, we have ‘Standardized’ with the equation [13], which makes the values centered around the mean values, including a unit standard deviation.

(1) $$X^{\prime }=\frac{X-\mu }{\sigma }$$

where μ is the mean and σ is the standard deviation.

### 2.3. Association and Impacts of the Features to the Patients

In this study, we have considered benign ovarian tumor patients as a control and the ovarian cancer patients as the case and then conducted two statistical analyses, including the Student’s *t*-test and the Mann–Whitney U-test, since it is suitable for finding the significant features for distinguishing patients’ benign ovarian tumors and ovarian cancer. For this analysis, we have used Statistical Package for the Social Sciences (SPSS), version 25.0. Significant features were chosen based on their *p*-values < 0.05. The Student’s *t*-test was used to analyze the association of the continuous variable attributes, where the features are reserved if they show significant correlation (i.e., *p*-value < 0.05); otherwise, they are omitted [14]. The Mann–Whitney U-test is used to compare two population means without the assumption of being drawn from standard distribution [15].

### 2.4. Machine Learning Models

In this study, we have used several supervised ensemble-based machine learning algorithms, including Logistic Regression (LR), Decision Tree (DT), Random Forest (RF), Light Gradient Boosting Machine (LGBM), Support Vector Machine (SVM), Extreme Gradient Boosting Machine (XGB), and Gradient Boosting Machine (GBM) separately to predict ovarian cancer, and we searched for the models with the best performance. We use grid search to fine-tune hyperparameters in our machine learning study, and we also employ five-fold cross-validation. For hyperparameter tuning, we have tuned ‘criterion’ as ‘gini’ and ‘entropy’, ‘max_depth’, ‘min_samples_split’, and ‘min_samples_leaf’ for DT, ‘max_depth’, ‘n_estimators’, and ‘learning_rate’ for XGB, ‘n_estimators’, ‘max_features’, ‘max_depth’, and ‘criterion’ for RF, ‘C’ and ‘gamma’ for SVM, ‘learning_rate’, ‘n_estimators’, ‘num_leaves’, ‘boosting_type’, ‘max_bin’, ‘colsample_bytree’, ‘subsample’, ‘reg_alpha’, ‘reg_alpha’ for LGBM, and ‘panalty’ and ‘C’ for LR, and ‘learning_rate’ and ‘min_samples_leaf’ for GBM algorithm.

For all ML studies, the Python (Python 3.7.13) programming language has been utilized. We have utilized Python libraries such as pandas and numpy for basic data processing and sklearn for machine learning. In addition, ‘matplotlib’ in Python and ‘ggplot2’ in the R programming language were applied to generate all plots and figures. For DT, XGB, RF, and GBM algorithms, we employed ‘feature_importance_’ to determine the importance of a feature; for SVM and LR, we applied the ‘coef_’ method; and for the LGBM algorithm, we invoked the ‘feature_importance()’ function.

Although the Random Forest (RF) algorithm performs classification tasks based on the majority voting of an ensemble of decision trees, to provide a fair and comprehensive comparison, we were interested to observe how RF outperforms a single decision tree prediction task.

Logistic Regression (LR) is a machine learning algorithm implemented in statistics used in binary classification problems. It intuits the maximum-likelihood value based on the best coefficient value [16]. In the logistic regression algorithm, we used the sigmoid function, which described the output as a number between 0 and 1. Finally, the threshold value was considered to classify the input dataset.

Decision Tree (DT) classifies samples by creating decision rules depending on the entropy function as well as the information gain [17], which can handle both the continuous and categorical data.

Random Forest (RF) makes use of several decision trees for classification, and its performance can be developed through accurately tuning the hyperparameter [18], which considers training data arbitrarily to handle the over-fitting problems in an efficient way [19]. In our analysis, the ‘gini’ function was used to measure the quality of splitting of the trees.

Support Vector Machine (SVM) makes a decision boundary to classify data and has been widely used in medical informatics. The ‘linear’ kernel is very commonly used in applications that employ SVM, where the *Cost* and *Gamma* are two of the controlling hyperparameters. The Cost parameter is used to handle the misclassification of training samples, and the Gamma parameter controls the decision region [18]. We have also used ‘linear’ kernel to find the feature importance. The Bayesian optimization method is used to optimize the parameter values of RBF to enhance classification performance.

Gradient Boosting Machine (GBM) is an ensemble learning method that merges multiple feeble learners to make a robust one through the optimization of the *loss* function [18] that normally uses the *deviance* or *exponential loss* function. Logistic regression is employed to handle deviance loss function, and *Adaboosting* is applied to control exponential loss function.

Light Gradient Boosting Machine (LGBM) is an improved version of GBM depending on tree-based learning techniques. It can potentially handle a massive volume of data and perform at a high-accuracy level with limited computing resources (i.e., memory space and computing speed) compared to other models [20]. The learning was tuned between (0.005, 0.01). The extreme gradient boosting (XGB) employs a gradient descent technique to diminish the loss while joining a new model. XGB supplies a boosting tree that resolves numerous data science problems with a fast and precise approach [18].

We have use Google Co-laboratory cloud platform to perform all the simulation tasks.

### 2.5. Evaluation Metrics

In this article, we have used several evaluation metrics, namely accuracy, precision, recall, F-score, AUC, and log-loss to evaluate the performances of the classifiers based on the True Positives (*TP*), False Positives (*FP*), True Negatives (*TN*) and True Positives (*TP*).

- Accuracy: Accuracy represents the correctness of a model [21], and it can be expressed as follows:
  (2) $$Accuracy=\frac{TP+TN}{TP+TN+FP+FN}$$
- Precision: Precision states the percentage of appropriately identified samples (positive) inside all identified samples (positive) [22], which can be stated as follows:
  (3) $$Precision=\frac{TP}{TP+FP}$$
- Recall: Recall expresses the capacity of the classifier to properly classify samples within a given class, which is as follows: [23]
  (4) $$Recall=\frac{TP}{TP+FN}$$
- F1-score: F1-score is used for the case when there is class imbalance in data by harmonizing Precision and Recall [22], which is as follows:
  (5) $$F1=\frac{2*Precision*Recall}{Precision+Recall}=\frac{2*TP}{2*TP+FP+FN}$$
- ROC-AUC: ROC-AUC denotes the discrimination capability of the model, and it shows the relationship between specificity and sensitivity [22].
  (6) $$Sensitivity=Recall=\frac{TP}{TP+FN}$$
  (7) $$Specificity=\frac{TN}{FP+TN}$$
  The Area Under the Curve (AUC) is the area under the Sensitivity(TRP)−(1−Specificity)(FPR) curve.
- Log-loss: Log-loss calculates the ambiguity of the probability of a method by analyzing them to the exact labels. A lesser log-loss value indicates improved predictions [24].
  (8) $$\begin{array}{c} H_{p}\left(q\right)=-\frac{1}{N}\sum_{i=1}^{N}y_{i}.log\left(p\left(y_{i}\right)\right)\\ +\left(1-y_{i}\right).log\left(1-p\left(y_{i}\right)\right) \end{array}$$
  Log loss is calculated as follows: H(q), where *y* is the level of the target variable, p(y) is the projected probability of the point given the target value, and *q* is the actual value of the log loss.

## 3. Results

In this study, the dataset consisted of 349 individual patients’ information, and there was only 7% missing values, which was handled by imputing the mean values. We also eliminated the entries that contained any missing values, and we found a total 106 patients’ data (44 benign tumors and 62 ovarian cancer tumors). The data-scaling technique was used to approximate mean values with standard deviation. We divided the whole dataset into 80% for training and 20% for testing. Accuracy, Precision, Recall, F1-score, AUC, and log-loss evaluation metrics are employed to test the classifier performance. We also implemented the Mann–Whitney U-test to detect the significant factors that are responsible for ovarian cancer.

### 3.1. Finding Significantly Associative Biomarkers Using Statistical Methods

**Figure 2:**
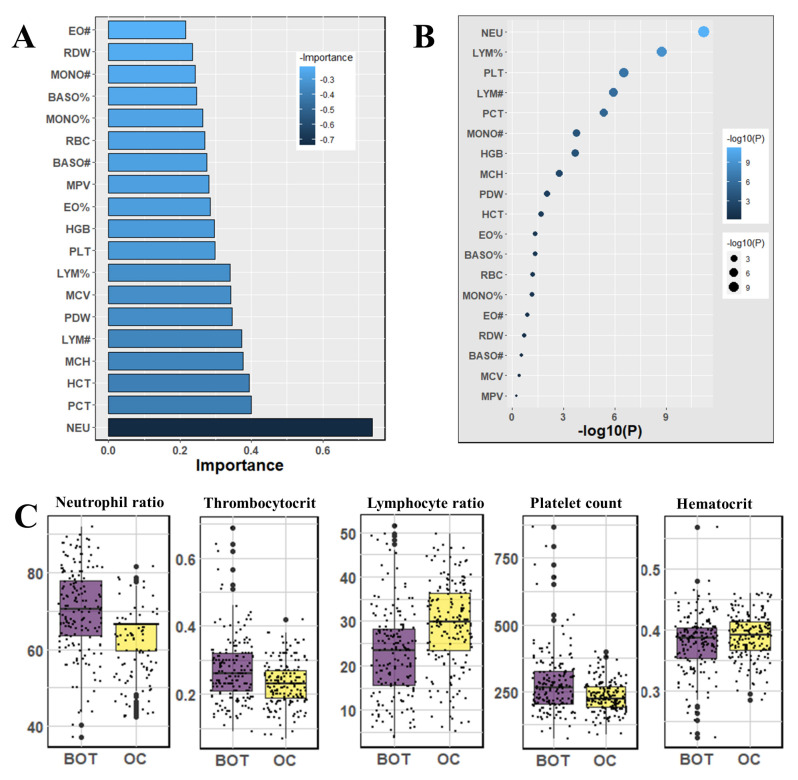
The analysis results for the dataset blood samples; (**A**) The feature importance of blood samples calculated by ML algorithms according to coefficient values after model training; (**B**) The association between benign ovarian tumor and ovarian cancer patients applying independent sample *t*-test, the lighter and larger bubble represent higher association; (**C**) The box plot of the five top most associated blood samples.

**Figure 3:**
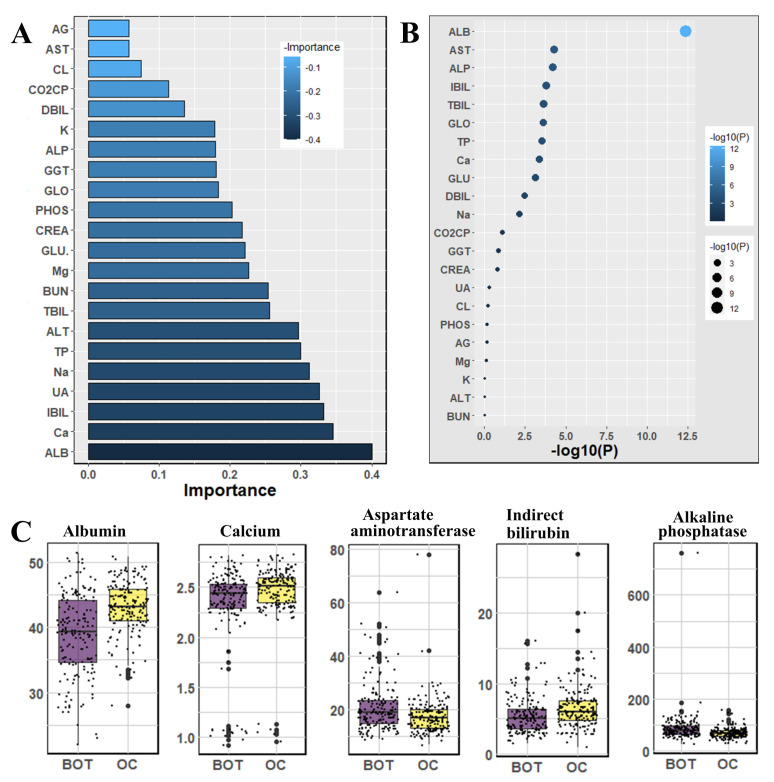
The analysis results for the dataset general chemistry tests; (**A**) The feature importance of general chemistry tests calculated by ML algorithms according to coefficient values after model training; (**B**) The association between benign ovarian tumor and ovarian cancer patients applying independent sample *t*-test, the lighter and larger bubble represent higher association; (**C**) The box plot of the five top most associated general chemistry tests.

**Figure 4:**
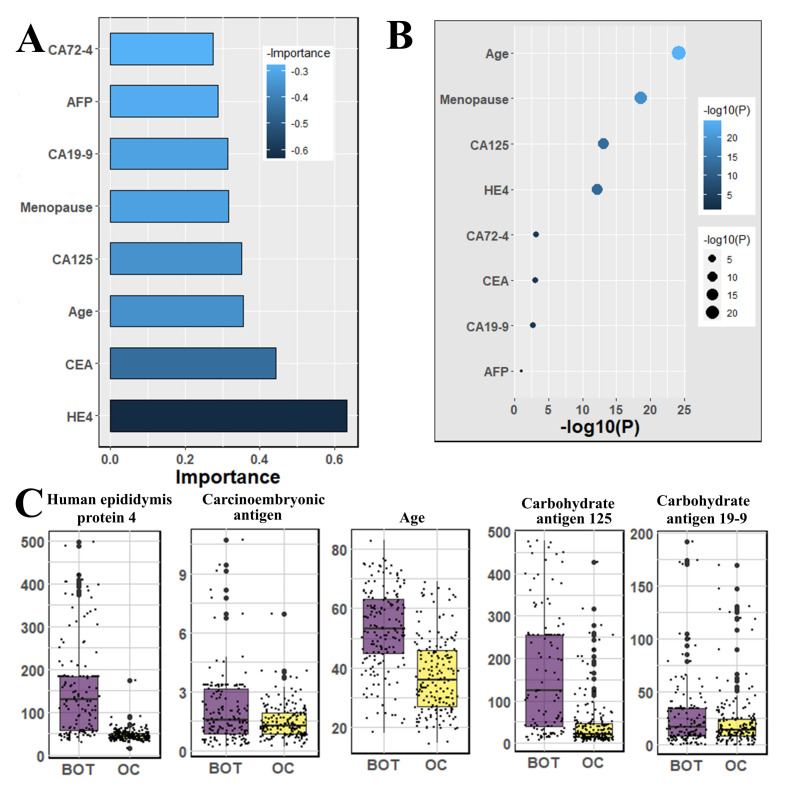
The analysis results for the dataset cancer markers; (**A**) The feature importance of cancer markers calculated by ML algorithms according to coefficient values after model training; (**B**) The association between benign ovarian tumor and ovarian cancer patients applying independent sample *t*-test, the lighter and larger bubble represent higher association; (**C**) The box plot of the four top most associated cancer markers with patients age.

We used the Mann–Whitney U-test and Student’s *t*-test to all datasets to identify important factors which are accountable for ovarian cancer. Our findings are shown in Figure 2, Figure 3 and Figure 4. The most significant descending order parameters are neutrophil ratio, lymphocyte ratio, platelet count, lymphocyte count, and thrombocytocrit in the blood sample dataset. The albumin, aspartate aminotransferase, alkaline phosphatase, indirect bilirubin, and globulin are the most critical attribute in descending order in the general chemistry dataset. The highest vital features in descending order are age, menopause, carbohydrate antigen 125, human epididymis protein 4, and carbohydrate antigen 72-4 in the OC marker dataset.

### 3.2. Classification of Ovarian Cancer Using Machine Learning Algorithms

In the case of the blood samples dataset, the highest Accuracy (82.0%), F1-score (83.0%), and AUC (82.0%) were calculated by GBM and LGBM. DT and RF performed the maximum precision of 83.0% and recall of 92.0%, respectively. The lowest log-loss value is 6.2, which LGBM manipulates. In the general chemistry dataset, RF showed the maximum accuracy (81.0%) and AUC (80.0%) and the minimum log-loss (6.71). LGBM calculated the highest precision 87.0% and F1-score 84.0%. However, SVM manipulated the peak recall, and it was 90.0%.

In the OC marker dataset, the highest accuracy (86.0%), recall (97.0%), AUC (86.0%), and the lowest log-loss (4.79) were evaluated by both RF and XGBoost classifiers. DT and RF showed the maximum precision (81.0%) and F1-score (87.0%), respectively. Other classifiers also achieved good results in all evaluation metrics. In the combined dataset, RF, GBM, and LGBM showed the maximum accuracy of 88.0%, AUC of 87.0%, and the minimum log-loss of 4.31. RF and GBM evaluated the highest recall of 95.00% and F1-score of 89.0%. LGBM demonstrated the uppermost precision, and it was 85.0%. Table 3 contains the results. Additionally, we determined the confusion matrices and displayed the outcomes in the Supplementary Table S1.

Additionally, we have removed any rows with any missing information, leaving us with a total of 106 patients’ data (44 benign tumor and 62 ovarian cancer). Table 4 contains the results. After comparing the outcomes of substituting missing values and removing missing values, it can be seen that the scores of the matrices differ significantly. With a few notable exceptions, such as the RF score of 0.91 accuracy for the OC marker dataset, almost of the scores of the dataset with the missing values removed were lower except for log-loss. In this situation, the missing values deleted scenario exhibits bad results because lower log-loss values suggest better results.

Our study also demonstrated the feature importance, and it is calculated depending on the average coefficient value of each used classifier. First, we determined the feature importance values for each algorithm, and then, we scaled the values using the min–max method to make them lie between 0 and 1. After that, we calculated the average values for each feature.

In Figure 3, we observed general chemistry’s feature importance and noticed the most significant feature is age. Other vital features are albumin, calcium, indirect bilirubin, uric acid, and so on. The least significant attributes are anion gap, aspartate aminotransferase, chlorine, carbon dioxide-combining power, direct bilirubin, and so on.

The highest rank feature is neutrophil ratio, and the lowest rank attribute is eosinophil count, respectively, in feature importance of blood routine (Figure 2). In the case of the feature importance of the tumor marker in Figure 4, the most and least significant features are human epididymis protein 4 and carbohydrate antigen 72-4, respectively.

## 4. Discussion

Early detection of ovarian cancer can reduce the rate of mortality by extending the survival life. Our analysis reveals three different ways of using a dataset to detect ovarian carcinomas in the early stage and finds different sets of biomarkers responsible for disease occurrence.

At first, the raw dataset was imputed for the missing entries followed by its normalization through scaling techniques. We divided the dataset into three parts based of different types of biomarkers, such as blood routine tests, general chemistry tests (serum), and tumor markers. We have applied statistical and machine learning methods individually over the grouped data. In the statistical analysis, we have identified the most significant biomarkers, whereas in machine learning classification approaches, we classify the patients in the two different groups, benign ovarian tumors and ovarian cancer, and we rank the features as important biomarkers according to their importance. Note that in the machine learning analysis, prior to the data scaling, firstly, we have split the whole data into two partitions: training and testing data following the ratio of 4:1. The tuning of model parameters was conducted based on the grid search technique using a five-fold cross-validation approach. After model training and cross-validation, we test the model with a test dataset and measure the accuracy, including evaluation matrices over the test dataset.

The usage of machine learning models is a broadly recognized mechanism for showing disease-related factors as distinguishing markers in predictive patient diagnostics [25,26]. Machine learning algorithms’ capacity to find hidden patterns in data by examining a collection of characteristics can lead to a better grasp of the understanding. The classification results with a higher accuracy score indicate reasonable prediction and ensure real-life applicability. Most models can predict accurately with above 80% accuracy score, precision, recall, F1-score, and AUC score. A low log-loss score in binary classification also justifies a good model performance. More specifically, RF, GBM, and LGBM models have achieved a comparatively good accuracy score compared with other matrices in some cases.

Our results suggest some important and significant biomarkers. Firstly, age and menopause are significant as demographic information. Some studies have also proven that menopause is a factor that is not directly responsible for ovarian cancer. However, most of the cases are diagnosed after menopause [27,28], because generally, menopause is not possible at an early age; ovarian cancer is detected after a certain age. In the analysis of Student’s *t*-test, we found the four most significant biomarkers, which were also identified by machine learning analysis, and they are carbohydrate antigen 125, carbohydrate antigen 19-9, carcinoembryonic antigen and human epididymis protein 4—all of those are serum samples. Among other significant biomarkers that we have found are: neutrophil ratio, thrombocytocrit, hematocrit as blood samples, alanine aminotransferase, calcium, indirect bilirubin, uric acid, and natrium as the general chemistry test.

Our analysis suggests that biomarkers are good enough to detect ovarian cancer, but a question could arise about which types of biomarkers are needed. In this study, we consider this question as well. The three different types of biomarkers (blood samples, general chemistry tests, and OC markers) are used differently and proved that those are capable to detect ovarian cancer separately. So, it will helpful with any set of biomarkers separately or in combination in the practical application to diagnose ovarian cancer.

We compare our work with previous work in Table 5 to prove the superiority of our research. Lu et al. [5] applied DT and obtained an accuracy of 87.00% and SE of 82.00%, respectively, in clinical data that contained 349 patients and 49 features. In another work, Akazawa and Hashimoto [12] used XGBoost to obtain an accuracy of 80.00%. In addition, Martinez-Mas [29] employed SVM and ELM classifiers and obtained an accuracy of 87.00% and SE of 87.00% and AUC of 89.00% in image data. In contrast, in our work, we achieved an accuracy of 88.00%, SE of 97.00%, and AUC of 87.00% using RF, GBM, and LGBM methods, which is better than the previous results. Furthermore, in our study, we have analyzed the individual datasets, i.e., blood samples, general chemistry tests (serum), and cancer biomarkers and combined data. Each of the analyses is individually capable of differing between benign tumor patients and malignant patients and identifying the most associative biomarkers using statistical methods.

This work was performed using a small amount of patients’ data for classification; thus, it is tough to make a generalized decision based on this study, though it could be a good enough predictive system in the use of the real-life application. Because most of the time, practitioners cannot detect cancer at an early stage, this system can help them diagnose in an early stage.

## 5. Conclusions

In this paper, we preprocessed the dataset and employed statistical and machine learning techniques to identify important features in early diagnosis of ovarian cancer patients. The most significant biomarkers accountable for ovarian cancer are carbohydrate antigen 125, carbohydrate antigen 19-9, carcinoem-bryonic antigen, and human epididymis protein 4. It also found that RF, GBM, and LGBM classifiers demonstrate a high degree of classification accuracy, which may be indicative that our work can be used for computer-aided clinical diagnostics to assist physicians and clinicians in analyzing ovarian cancer in a low-cost manner. Another important implication of our work is to reduce cancer identification time. The main limitation of our research was the amount of data. In the future, we will use more data to explore ovarian cancer including the control group of patients.

## Figures and Tables

**Table 1:** The attribute list for different subgroups of the dataset.

Blood Routine Test	General Chemistry	Tumor Marker
Neutrophil ratio	Albumin	Carbohydrate antigen 72-4
Thrombocytocrit	Indirect bilirubin	Alpha-fetoprotein
Hematocrit	Uric acid	Carbohydrate antigen 19-9
Mean corpuscular hemoglubin	Nutrium	Menopause
Lymphocyte	Total protein	Carbohydrate antigen 125
Platelet distribution width	Alanine aminotransderase	Carcinoembryonic antigen
Mean corpuscular volume	Total bilirubin	Age
Platelet count	Blood urea nitrogen	Human epididymic protein 4
Hemoglobin	Magnesium	
Eosinophil ratio	Glucose	
Mean platelet volume	Creatinine	
Basophil cell count	Phosphorus	
Red blood cell count	Globulin	
Mononuclear cell count	Gama glutamyl tranferasey	
Red blood cell distribution width	Alkaline phosphates	
Basophil cell ratios	Kalium	
	Direct bilirubin	
	Carban dioxide-combining power	
	Chlorine	
	Aspartate aminotransferase	
	Anion gap	

**Table 2:** Association between benign ovarian tumor and ovarian cancer patients. The results of independent sample *t*-test with blood samples, general biochemistry tests and tumor markers. N.B. BOT: Benign Ovarian Tumor; OC: Ovarian Cancer; SD: Standard Deviation.

Abbreviation	Biomarkers	Type	Unit	Mean ± SD		95% CI	*p*
				BOT	OC		
MPV	Mean platelet volume	full blood	fL	9.98±1.78	10.09±1.69	(−0.48, 0.25)	0.55
BASO#	Basophil cell count	full blood	$10^{9}$/L	0.28±0.02	0.03±0.02	(−0.006, 0.002)	0.28
PHOS	Phosphorus	serum	mmol/L	1.12±0.18	1.12±0.19	(−0.05, 0.03)	0.67
GLU	Glucose	serum	mmol/L	5.56±1.18	5.12±0.85	(0.18, 0.69)	<0.01
CA72-4	Carbohydrate antigen 72-4	serum	U/mL	12.77±19.32	7.67±4.15	(2.18, 8.01)	<0.01
K	Kalium	serum	mmol/L	4.38±0.41	4.39±0.39	(−0.4, −1.17)	0.92
AST	Aspartate aminotransferase	serum	u/L	20.94±9.36	17.34±6.88	(1.87, 5.32)	<0.01
BASO%	Basophil cell ratio	full blood	%	0.44±0.33	0.52±0.36	(−0.15, −0.001)	0.05
Mg	Magnesium	serum	mmol/L	0.98±0.13	0.98±0.12	(−0.03, 0.02)	0.78
CL	Chlorine	serum	mmol/L	100.9±3.52	100.73±2.36	(−0.05, 1.07)	0.6
CEA	Carcinoembryonic antigen	serum	ng/mL	5.23±15.02	1.47±0.88	(1.55, 5.98)	<0.01
EO#	Eosinophil count	full blood	$10^{9}$/L	0.06±0.06	0.07±0.08	(−0.03, 0.003)	0.13
CA19-9	Carbohydrate antigen 19-9	serum	U/mL	66.66±166.39	25.91±40.36	(15.48, 66.01)	<0.01
ALB	Albumin	serum	g/L	38.94±6.27	43.13±3.92	(−5.29, −3.1)	<0.01
IBIL	Indirect bilirubin	serum	umol/L	5.36±2.51	6.54±3.15	(−1.77, −0.57)	<0.01
GGT	Gama glutamyl transferase	serum	u/L	22.79±17.22	19.88±18.48	(−0.85, 6.68)	0.13
MCH	Mean corpuscular hemoglobin	full blood	Pg	28.34±2.71	29.2±2.36	(−1.39, −0.32)	<0.01
GLO	Globulin	serum	g/L	31.07±5.04	29.32±3.69	(0.83, 2.68)	<0.01
ALT	Alanine aminotransferase	serum	u/L	17.96±11.79	18.06±10.39	(−2.44, 2.23)	0.93
DBIL	Direct bilirubin	serum	umol/L	2.9±1.34	3.35±1.45	(−0.74, −0.15)	<0.01
RDW	Red blood cell distribution width	full blood	%	13.68±2.0	13.43±1.59	(−0.13, 0.62)	0.2
PDW	Platelet distribution width	full blood	%	13.91±3.19	14.74±2.74	(−1.46, −0.21)	<0.01
CREA	Creatinine	serum	umol/L	63.35±12.59	65.11±10.78	(−4.23, 0.7)	0.16
AFP	Alpha-fetoprotein	serum	ng/mL	20.41±135.44	2.72±2.18	(−2.28, 37.66)	0.08
HGB	Hemoglobin	full blood	g/L	122.21±16.71	128.34±13.7	(−9.35, −2.93)	<0.01
Na	Natrium	serum	mmol/L	140.91±3.26	140.1±2.35	(0.22, 1.42)	<0.01
HE4	Human epididymis protein 4	serum	pmol/L	324.24±488.62	49.17±39.07	(202.8, 347.34)	<0.01
LYM#	Lymphocyte count	full blood	$10^{9}$/L	1.41±0.54	1.7±0.55	(−0.4, −0.17)	<0.01
CA125	Carbohydrate antigen 125	serum	U/mL	652.15±1021.91	51.45±79.79	(449.57, 751.81)	<0.01
BUN	Blood urea nitrogen	serum	mmol/L	4.0±1.42	4.02±1.15	(−0.28, 0.26)	0.94
LYM%	Lymphocyte ratio	full blood	%	22.74±10.05	29.27±9.7	(−8.61, −4.46)	<0.01
Ca	Calcium	serum	mmol/L	2.32±0.42	2.46±0.28	(−0.21, −0.06)	<0.01
AG	Anion gap	serum	mmol/L	19.4±4.6	19.24±4.09	(−0.75, 1.08)	0.73
MONO#	Mononuclear cell count	full blood	$10^{9}$/L	0.39±0.16	0.33±0.13	(−0.03, 0.78)	<0.01
PLT	Platelet count	full blood	$10^{9}$/L	281.65±117.44	230.25±57.33	(32.06, 70,74)	<0.01
NEU	Neutrophil ratio	full blood	%	70.21±10.92	63.09±7.5	(5.16, 9.09)	<0.01
EO%	Eosinophil ratio	full blood	$10^{9}$/L	1.0±0.94	1.24±1.29	(−0.48, −0.004)	0.05
TP	Total protein	serum	g/L	69.66±8.78	72.45±5.07	(−4.29, −1.29)	<0.01
UA	Uric acid	serum	μmol/L	246.25±78.04	241.26±58.18	(−9.46, 19.45)	0.5
RBC	Red blood cell count	full blood	$10^{12}$/L	4.31±0.52	4.41±0.41	(−0.19, 0.005)	0.06
PCT	Thrombocytocrit	full blood	L/L	0.27±0.1	0.23±0.07	(0.02,0.06)	<0.01
CO2CP	Carban dioxide-combining power	serum	mmol/L	24.54±2.97	24.03±2.36	(−0.05, 1.07)	0.08
TBIL	Total bilirubin	serum	μmol/L	8.27±3.6	9.88±4.4	(−2.46, −0.77)	<0.01
HCT	Hematocrit	full blood	L/L	0.38±0.05	0.39±0.04	(−0.02, −0.002)	0.02
MONO%	Monocyte ratio	full blood	%	5.77±2.01	5.4±1.82	(−0.03, 0.78)	0.07
MCV	Mean corpuscular volume	full blood	fL	87.79±6.71	88.34±5.32	(−1.82, 0.72)	0.4
ALP	Alkaline phosphatase	serum	u/L	86.56±57.77	67.98±19.61	(9.56, 27.59)	<0.01

**Table 3:** Accuracy and evaluation matrices scores for each of the data groups.

Dataset	Model	Accuracy	Precision	Recall	F1-Score	AUC	Log Loss
Blood Samples	RF	0.81	0.76	0.92	0.82	0.78	7.6
	SVM	0.81	0.77	0.89	0.82	0.78	7.8
	DT	0.81	0.83	0.78	0.81	0.81	6.71
	XGBoost	0.81	0.78	0.86	0.82	0.77	7.6
	LR	0.80	0.79	0.81	0.80	0.78	7.6
	GBM	0.82	0.82	0.84	0.83	0.82	6.23
	LGBM	0.82	0.80	0.86	0.83	0.82	6.2
General Chemistry	RF	0.81	0.80	0.83	0.82	0.80	6.71
	SVM	0.80	0.76	0.90	0.81	0.79	7.11
	DT	0.68	0.70	0.68	0.69	0.68	11.03
	XGBoost	0.76	0.76	0.78	0.78	0.77	8.15
	LR	0.80	0.75	0.89	0.82	0.79	7.11
	GBM	0.75	0.76	0.76	0.76	0.75	8.63
	LGBM	0.75	0.87	0.82	0.84	0.76	7.11
OC Marker	RF	0.86	0.80	0.97	0.87	0.86	4.79
	SVM	0.85	0.80	0.95	0.86	0.84	5.27
	DT	0.85	0.81	0.92	0.86	0.85	5.2
	XGBoost	0.86	0.80	0.97	0.86	0.86	4.79
	LR	0.83	0.80	0.92	0.85	0.83	5.7
	GBM	0.85	0.80	0.95	0.86	0.84	5.27
	LGBM	0.85	0.80	0.95	0.86	0.84	5.27
Combined	RF	0.88	0.83	0.95	0.89	0.87	4.31
	SVM	0.81	0.77	0.89	0.83	0.80	6.71
	DT	0.78	0.78	0.78	0.78	0.78	7.6
	XGBoost	0.86	0.82	0.95	0.86	0.86	4.79
	LR	0.82	0.79	0.89	0.84	0.82	6.23
	GBM	0.88	0.83	0.95	0.89	0.87	4.31
	LGBM	0.88	0.85	0.92	0.88	0.87	4.31

**Table 4:** Accuracy and evaluation matrices scores for each of the data groups for the dataset of 106 patients.

Dataset	Model	Accuracy	Precision	Recall	F-1 Score	AUC	Log-Loss
Blood Samples	RF	0.86	0.82	1	0.9	0.81	4.71
	SVM	0.81	0.77	1	0.88	0.75	6.28
	DT	0.77	0.8	0.86	0.83	0.74	7.85
	XGBoost	0.77	0.8	0.86	0.83	0.74	7.85
	LR	0.82	0.78	1	0.88	0.75	6.28
	GBM	0.73	0.72	0.73	0.72	0.68	9.42
	LGBM	0.64	0.64	1	0.78	0.5	12.56
General Chemistry	RF	0.77	0.76	0.93	0.84	0.71	7.85
	SVM	0.77	0.76	0.93	0.84	0.71	7.85
	DT	0.59	0.67	0.71	0.69	0.54	14.13
	XGBoost	0.73	0.75	0.86	0.8	0.68	9.42
	LR	0.77	0.76	0.93	0.84	0.71	7.85
	GBM	0.73	0.72	0.73	0.72	0.68	9.42
	LGBM	0.64	0.64	1	0.78	0.5	12.56
OC Marker	RF	0.91	1	0.86	0.92	0.93	3.14
	SVM	0.82	0.92	0.79	0.85	0.83	6.28
	DT	0.59	1	0.36	0.53	0.68	14.13
	XGBoost	0.68	1	0.5	0.67	0.75	10.99
	LR	0.82	0.92	0.79	0.85	0.83	6.28
	GBM	0.81	0.84	0.82	0.82	0.83	6.28
	LGBM	0.64	0.64	1	0.78	0.5	12.56
Combined	RF	0.86	0.87	0.93	0.9	0.84	4.71
	SVM	0.64	0.8	0.57	0.67	0.66	12.56
	DT	0.68	1	0.5	0.67	0.75	10.99
	XGBoost	0.86	1	0.79	0.88	0.89	4.71
	LR	0.86	0.82	1	0.9	0.81	4.71
	GBM	0.86	0.87	0.86	0.87	0.87	4.71
	LGBM	0.64	0.64	1	0.78	0.5	12.56

**Table 5:** A comparison between proposed methods and previous methods.

References	Dataset	Classifiers	Accuracy	Sensitivity	AUC
[5]	Clinical data (349 patients with 49 features)	DT	0.87	0.82	-
[12]	Clinical data (202 patients with 32 features)	XGBoost	0.80	-	-
[29]	Image data (348 patients)	SVM, ELM	0.87	0.87	0.89
Proposed	Clicnical data (349 patients with 49 features)	RF, GBM, LGBM	0.88	0.97	0.87
Proposed	Clicnical data (106 patients with OC marker features)	RF	0.91	0.86	0.93

## Data Availability

The dataset and corresponding codes are available in the following repository: https://github.com/martuzaiu/Ovarian_Cancer_Project (accessed date: 1 July 2022).

## Supplementary Materials

The following are available online at https://www.mdpi.com/article/10.3390/jpm12081211/s1, Supplementary Table S1: Confusion matrics of the machine learning algorithms.

## Abbreviations

The following abbreviations are used in this manuscript:

MPV	Mean platelet volume
BASO#	Basophil cell count
PHOS	Phosphorus
GLU.	Glucose
CA72-4	Carbohydrate antigen 72-4
K	Kalium
AST	Aspartate aminotransferase
BASO%	Basophil cell ratio
Mg	Magnesium
CL	Chlorine
CEA	Carcinoembryonic antigen
EO#	Eosinophil count
CA19-9	Carbohydrate antigen 19-9
ALB	Albumin
IBIL	Indirect bilirubin
GGT	Gama glutamyltransferasey
MCH	Mean corpuscular hemoglubin
GLO	Globulin
ALT	Alanine aminotransferase
DBIL	Direct bilirubin
RDW	Red blood cell distribution width
PDW	Platelet distribution width
CREA	Creatinine
AFP	Alpha-fetoprotein
HGB	Hemoglobin
Na	Natrium
HE4	Human epididymis protein 4
LYM#	Lymphocyte count
CA125	Carbohydrate antigen 125
BUN	Blood urea nitrogen
LYM%	Lymphocyte ratio
Ca	Calcium
AG	Anion gap
MONO#	Mononuclear cell count
PLT	Platelet count
NEU	Neutrophil ratio
EO%	Eosinophil ratio
TP	Total protein
UA	Uric acid
RBC	Red blood cell count
PCT	Thrombocytocrit
$CO_{2}CP$	Carban dioxide-combining power
TBIL	Total bilirubin
HCT	Hematocrit
MONO%	Monocyte ratio
MCV	Mean corpuscular volume
ALP	Alkaline phosphatase
