# Supplementary material for: Early-Stage Detection of Ovarian Cancer Based on Clinical Data Using Machine Learning Approaches
Source: J Pers Med. 2022 Jul 25;12(8):1211. doi: 10.3390/jpm12081211 (PMC9394434; doi:10.3390/jpm12081211)
Supplement: Supplementary file 1 [file jpm-12-01211-s001.zip › jpm-1810777-supplementary.pdf]

Table S1. Confusion matrics of the machine learning algorithms

A. Combined Dataset

DT

Training			Testing		
	False Negative	False Positive		False Negative	False Positive
True Negative	143	0	True Negative	27	8
True Positive	1	140	True Positive	8	29

XGB

Training			Testing		
	False Negative	False Positive		False Negative	False Positive
True Negative	143	0	True Negative	27	8
True Positive	0	141	True Positive	2	35

RF

Training			Testing		
	False Negative	False Positive		False Negative	False Positive
True Negative	140	3	True Negative	28	7
True Positive	0	141	True Positive	2	35

SVM

Training			Testing		
	False Negative	False Positive		False Negative	False Positive
True Negative	132	11	True Negative	25	10
True Positive	2	139	True Positive	4	33

LR

Training			Testing		
	False Negative	False Positive		False Negative	False Positive
True Negative	135	8	True Negative	26	9
True Positive	6	135	True Positive	4	33

LGBM

Training			Testing		
	False Negative	False Positive		False Negative	False Positive
True Negative	128	15	True Negative	29	6
True Positive	6	135	True Positive	3	34

GBM

Training			Testing		
	False Negative	False Positive		False Negative	False Positive
True Negative	143	0	True Negative	28	7
True Positive	0	141	True Positive	2	35

B. Blood Sample Dataset

DT

Training			Testing		
	False Negative	False Positive		False Negative	False Positive
True Negative	134	9	True Negative	29	6
True Positive	3	138	True Positive	8	29

XGB

Training			Testing		
	False Negative	False Positive		False Negative	False Positive
True Negative	126	17	True Negative	26	9
True Positive	20	121	True Positive	5	32

RF

Training			Testing		
	False Negative	False Positive		False Negative	False Positive
True Negative	140	3	True Negative	24	11
True Positive	0	141	True Positive	3	34

SVM

Training			Testing		
	False Negative	False Positive		False Negative	False Positive
True Negative	109	34	True Negative	25	10
True Positive	23	118	True Positive	4	33

LR

Training			Testing		
	False Negative	False Positive		False Negative	False Positive
True Negative	109	34	True Negative	27	8
True Positive	25	116	True Positive	7	30

LGBM

Training			Testing		
	False Negative	False Positive		False Negative	False Positive
True Negative	127	16	True Negative	27	8
True Positive	22	119	True Positive	6	31

GBM

Training			Testing		
	False Negative	False Positive		False Negative	False Positive
True Negative	143	0	True Negative	28	7
True Positive	0	141	True Positive	6	31

C. General Chemistry Test Dataset

DT

Training			Testing		
	False Negative	False Positive		False Negative	False Positive
True Negative	138	5	True Negative	24	11
True Positive	8	133	True Positive	12	25

XGB

Training			Testing		
	False Negative	False Positive		False Negative	False Positive
True Negative	138	5	True Negative	26	9
True Positive	4	137	True Positive	8	29

RF

Training			Testing		
	False Negative	False Positive		False Negative	False Positive
True Negative	135	8	True Negative	27	8
True Positive	3	138	True Positive	6	31

SVM

Training			Testing		
	False Negative	False Positive		False Negative	False Positive
True Negative	118	25	True Negative	24	11
True Positive	16	125	True Positive	4	33

LR

Training			Testing		
	False Negative	False Positive		False Negative	False Positive
True Negative	119	24	True Negative	24	11
True Positive	19	122	True Positive	4	33

LGBM

Training			Testing		
	False Negative	False Positive		False Negative	False Positive
True Negative	138	5	True Negative	33	2
True Positive	71	70	True Positive	23	14

GBM

Training			Testing		
	False Negative	False Positive		False Negative	False Positive
True Negative	142	1	True Negative	28	9
True Positive	0	141	True Positive	9	26

D. OC Marker Dataset

DT					
Training			Testing		
	False Negative	False Positive		False Negative	False Positive
True Negative	136	7	True Negative	27	8
True Positive	7	134	True Positive	3	34

XGB					
Training			Testing		
	False Negative	False Positive		False Negative	False Positive
True Negative	132	11	True Negative	26	9
True Positive	2	139	True Positive	1	36

RF					
Training			Testing		
	False Negative	False Positive		False Negative	False Positive
True Negative	128	15	True Negative	26	9
True Positive	1	140	True Positive	1	36

SVM					
Training			Testing		
	False Negative	False Positive		False Negative	False Positive
True Negative	122	21	True Negative	26	9
True Positive	5	136	True Positive	2	35

LR					
Training			Testing		
	False Negative	False Positive		False Negative	False Positive
True Negative	123	9	True Negative	26	9
True Positive	7	134	True Positive	3	34

LGBM					
Training			Testing		
	False Negative	False Positive		False Negative	False Positive
True Negative	129	14	True Negative	26	9
True Positive	12	129	True Positive	5	32

GBM					
Training			Testing		
	False Negative	False Positive		False Negative	False Positive
True Negative	142	1	True Negative	26	9
True Positive	0	141	True Positive	2	35
